# Adolescents’ Emotion Regulation Strategies Questionnaire: Initial Validation and Prospective Associations With Nonsuicidal Self-Injury and Other Mental Health Problems in Adolescence and Young Adulthood in a Swedish Youth Cohort

**DOI:** 10.3389/fpsyt.2020.00462

**Published:** 2020-05-28

**Authors:** Ya Zhou, Daiva Daukantaitė, Lars-Gunnar Lundh, Margity Wångby-Lundh, Adam Ryde

**Affiliations:** Department of Psychology, Lund University, Lund, Sweden

**Keywords:** emotion regulation, adolescence, nonsuicidal self-injury, young adulthood, longitudinal

## Abstract

Although there is extensive research indicating the vital role of functional emotion regulation (ER) in healthy psychological development, such research has neglected examination of adolescents. One potential reason for this neglect is the lack of valid ER instruments developed specifically for adolescents. Further, the available ER instruments for adolescents usually require elaborate forms of cognitive reasoning about the internal sequences of cognitions and emotions. To address these limitations, we developed the Adolescents’ Emotion Regulation Strategies Questionnaire (AERSQ), a self-report instrument of adolescents’ commonly used ER strategies in daily life and examined its psychometric characteristics in a 10-year, three-wave cohort of Swedish youths (original N = 991, mean age = 13.7, 14.8, and 25.3 at waves 1, 2, and 3, respectively). Exploratory (wave 1 data) and confirmatory (wave 2 data) factor analyses revealed a five-factor structure for the AERSQ: rumination/negative thinking, positive reorientation, communication, distraction, and cultural activities. We observed gender differences for most ER strategies in adolescence. We also evaluated the associations between the AERSQ subscales and mental health (self-harm; psychological difficulties including hyperactivity, conduct problems, emotional problems, and peer problems; prosocial behavior; depression; anxiety; stress; flourishing; and life satisfaction) across the three time points. Rumination/negative thinking had the strongest relationships with these mental health indicators, both cross-sectionally and longitudinally, in both genders. Distraction and cultural activities were less related to mental health, especially prospectively. Although the AERSQ showed good test–retest reliability and predictive validity over a 10-year period, the low internal consistency of two of its subscales (distraction and cultural activities) indicates that it may benefit from further development both in terms of the included items and psychometric testing.

## Introduction

Over the last few decades, emotion regulation (ER) has occupied an increasingly important position in psychology and related fields. Research on ER can be traced back to psychoanalytic work on defense mechanisms (1). Although there remains no consensus on the definition of ER, it generally refers to “all the extrinsic and intrinsic processes responsible for monitoring, evaluating and modifying emotional reactions, especially their intensive and temporal features, to accomplish one’s goal” [(2), pp.27–28]. Theoretically, ER processes involve not only the down-regulation of negative emotions, but also the preservation or up-regulation of positive emotions (3). In daily life, however, ER usually targets negative emotions (4). Children from a very young age learn how to regulate their emotions in effective and socially appropriate ways, and this ability further develops throughout adolescence and adulthood. There is extensive literature indicating the vital role of functional ER in individuals’ mental and physical health and wellbeing, as well as its close relations with cognitive, behavioral, and social functioning and personality development (5). However, not everyone develops functional ER, and an increasing number of studies suggest that emotion dysregulation is an underlying mechanism of a number of psychiatric disorders (*e.g.*, depression, anxiety, substance abuse, eating disorder, borderline personality disorder) (6, 7).

Emotion dysregulation is also closely associated with nonsuicidal self-injury (NSSI), which is defined as the direct, deliberate destruction of one’s own bodily tissue (*e.g.*, cutting, burning, carving) without an intent to die (8). According to the four-function model of NSSI (8), NSSI is maintained *via* four reinforcement processes: intrapersonal negative reinforcement (*i.e.*, the NSSI decreases or distracts the individual from aversive thoughts or feelings), intrapersonal positive reinforcement (*i.e.*, the NSSI generates desired feelings or stimulation), interpersonal positive reinforcement (*i.e.*, the NSSI facilitates help-seeking), or interpersonal negative reinforcement (*i.e.*, the NSSI facilitates escape from undesired social situations). The two intrapersonal functions may be combined into a single function representing emotion dysregulation (9). Indeed, according to the ER model of NSSI (10–12), self-injury may serve an ER function, with the physical pain being used to reduce emotional pain. It is assumed that in the absence of more functional forms of ER, individuals experiencing severe emotional pain may resort to NSSI.

The majority of existing ER measures have been developed for use with adults and young children. Considering that NSSI and most psychiatric disorders have their initial onset in adolescence, there is a considerable need for valid measures of ER targeted towards adolescents. Thus, the main purpose of this study is to examine the usefulness and psychometric features of a self-report measure of adolescents’ ER strategies developed as part of the first phase of a 10-year longitudinal research project featuring a large sample of Swedish adolescents. We also sought to elucidate the prospective associations between adolescents’ ER strategies, their self-injurious behaviors, and other mental health problems in adolescence and young adulthood.

### Conceptualization of ER

The empirical conceptualization and measurement of ER has generally followed two distinct approaches. The first approach emphasizes individual variations in the habitual utilization of strategies for regulating emotions, whereas the other approach focuses on dispositional emotion regulation abilities. Regarding the first approach, some frequently studied ER strategies include acceptance, problem solving, reappraisal, mindfulness, distraction, rumination, expressive suppression, behavioral avoidance, and experiential avoidance (13). These strategies can be classified as adaptive or maladaptive. The former refer to strategies (*e.g.*, acceptance, problem solving, reappraisal, mindfulness) generally evidenced to have associations with adaptive outcomes such as improved psychological functioning and well-being and diminished psychopathology, while the latter (*e.g.* rumination, expressive suppression, behavioral avoidance, and experiential avoidance) are strategies linked with more maladaptive outcomes (14). Arguably, these qualities (adaptive and maladaptive) are most meaningful when applied to the individual ER process as a whole, and less so when attributed to specific ER strategies. As posited by Aldao (15), any strategy can be adaptive or maladaptive depending on the person, context, and goal, and utilizing strategies flexibly to match the context might be more crucial for successful ER than utilizing only the putatively adaptive strategies and not the putatively maladaptive strategies.

Classifications of ER strategies have utilized other models and dimensions other than the adaptive/maladaptive one. Gross’s process model of ER (3, 16) is probably the most influential model in ER research. The model specifies four stages in the temporal sequence of the emotion formation process: 1) a situation (real or imagined) that is emotionally relevant; 2) attention towards the emotional situation; 3) evaluation and interpretation of the emotional situation in light of one’s current goal; and 4) generation of an emotional response (comprising experiential, behavioral, and physiological components). According to this model, each of these four stages is subject to regulation, with the specific ER strategies being differentiated as antecedent-focused, which are strategies used before the complete activation of an emotion response (*e.g.*, situation selection or modification, attentional deployment, cognitive reappraisal), or as response-focused, which are strategies used after an emotion has been experienced (*e.g.*, experiential avoidance, expressive suppression).

ER strategies can also be classified as cognitive (*e.g.*, reappraisal, rumination) and behavioral (*e.g.*, eating, drinking alcohol, exercise) (17). Another important dimension to understanding the nature of a specific ER strategy is whether internal/intrapersonal or external/interpersonal resources are used for regulating emotions (18). Most of the frequently investigated strategies are considered internal/intrapersonal (*e.g.*, rumination, reappraisal, acceptance, mindfulness). In contrast, there is less empirical work on external/interpersonal strategies. However, such strategies are also essential for ER, since children typically develop their ER in a social context and it remains inextricably intertwined with their social relations throughout the life span (19).

The second common approach to conceptualizing and measuring ER emphasizes dispositional emotion regulation abilities (*e.g.*, emotional clarity, distress tolerance, impulse control), which are regarded as indicative of one’s ER potential. The Affect Regulation Training (ART) model (20) proposes that the interaction of multiple skills (*e.g.*, being aware of emotions, able to identify and label emotions, able to actively modify negative emotions in order to feel better, resilient and able to tolerate negative emotions, able to confront emotionally distressing situations in order to attain one’s goals) in specific emotional situations helps to facilitate the development of adaptive ER abilities.

### Measurement of ER

As stated above, numerous ER measures have been developed for adults. For the research approach emphasizing strategies, there are, for example, the Emotion Regulation Questionnaire [ERQ; (21)], the Cognitive Emotion Regulation Questionnaire [CERQ; (22)], the Interpersonal Emotion Regulation Questionnaire [IERQ; (23)], and the Ruminative Response Scale [RRS; (24)]. As for the approach emphasizing abilities, example measures include the Difficulties in Emotion Regulation Scale [DERS; (25)] and the Emotion-Regulation Skills Questionnaire [SEK-27, based on the ART model; (26)].

Some measures developed for adults have been adapted and validated for young people. For example, Gullone and Taffe (27) made the first attempt to adapt the ERQ for use with children and adolescents, Garnefski et al. (22) initially validated the adolescent version of the CERQ, while Burwell and Shirk (28) adapted the RRS for use with adolescents. An adolescent version of the DERS was created and validated by Weinberg and Klonsky (29). Later, Kaufman et al. (30) developed a short-form adolescent version of the DERS to reduce respondent burden. These adapted measures are now in wide use, but are restricted by the number or variety of ER strategies/abilities measured. Specifically, the ERQ assesses only two specific ER strategies (*i.e.* positive reappraisal and expressive suppression), the CERQ focuses solely on cognitive ER strategies, and the DERS exclusively measures difficulties in ER. Another important limitation of these adapted measures is that since they were originally designed for adults, their items might not give full consideration to the distinctive attributes of ER in adolescents; as such, they cannot be expected to provide a comprehensive measurement of adolescent ER.

Research has shown that the brain regions involved in the generation and regulation of emotions undergo protracted structural and functional development during adolescence (31). Specifically, the executive functions needed for ER, including working memory, inhibitory control, abstract thought, and decision making, all undergo development during these years (32). As such, it is imperative to develop age-relevant instruments for the measurement of ER in adolescents.

There are some ER measures originally developed for use with children, such as the Children’s Emotion Management Scale [CEMS; (33)], and the Emotion Regulation Checklist [ERC; (34)]. However, these measures have not been validated for use with adolescents. Moreover, measures designed for children typically utilize parents or other adults as informants, and while other-report ER measures may be suitable for young children, they are likely to be inappropriate for use with adolescents, given that adolescents are cognitively more mature and others may not be fully aware of adolescents’ ER processes. A self-report measure would therefore be a more appropriate method for measuring adolescents’ ER.

Very few ER measures have been originally developed for and validated with adolescents. First, Phillips and Power (35) developed the Regulation of Emotions Questionnaire (REQ), a 20-item self-report measure of four types of ER strategies used by adolescents in daily life: functional-intrapersonal, functional-interpersonal, dysfunctional-intrapersonal, and dysfunctional-interpersonal. Unfortunately, this measure has yet to be extensively validated. Phillips and Power (35) examined the psychometric properties of the REQ in a small sample of 225 adolescents (12–19 years), conducting both exploratory and confirmatory factor analyses on the same sample. Another measure is the Questionnaire to Assess Children’s and Adolescents’ Emotion Regulation Strategies (FEEL-KJ). The FEEL-KJ, originally written in German (36), is a 90-item self-report measure assessing 15 ER strategies (*i.e.*, problem-oriented action, cognitive problem-solving, acceptance, forgetting, distraction, revaluation, humor enhancement, giving up, withdrawal, rumination, self-devaluation, aggressive action, social support, expression, and emotional control) in response to anxiety, sadness, and anger (30 items each). Cracco, Van Durme, and Braet (37) tested the reliability and validity of the FEEL-KJ in a large sample of Dutch-speaking Belgian children and adolescents (N = 1,102, 8–18 years). They confirmed the two-factor structure of the original, with adaptive and maladaptive ER serving as overarching categories. However, it remained unclear how the social support, expression, and emotional control strategies fit within the FEEL-KJ structure. Thus, although the FEEL-KJ assesses a broad variety of ER strategies, its internal structure remains to be clarified. Moreover, the Responses to Stress Questionnaire (RSQ), a measure developed by Connor-Smith et al. (38) for assessing adolescents’ controlled coping and automatic responses to stress, also includes some items representing ER strategies (*e.g.*, rumination, problem solving, distraction, avoidance).

An important drawback of existing instruments for measuring ER, whether developed for adults or for adolescents, is that they often contain items that require rather elaborate forms of cognitive reasoning about the internal sequences of cognitions and emotions. For example, Gross and John’s (21) ERQ contains items like “I control my emotions by changing the way I think about the situation I’m in,” which requires the ability to reason about the sequential relations between cognitions and emotions on the basis of self-observation, and about how to change one’s thinking to produce emotional change. Gratz and Roehmer’s (25) DERS similarly contains items like “When I’m upset, I feel guilty for feeling that way,” which require the ability to reason about the relation between stressful feelings and second-order self-conscious emotions. Similarly, although Garnefski et al.’s (22) CERQ and Phillips and Power’s (35) REQ have been successfully applied to adolescents, they both contain items requiring meta-cognitive reasoning about general tendencies in one’s way of handling emotional experiences (*e.g.*, the CERQ item “I am preoccupied with what I think and feel about what I have experienced” or the REQ item “I take my feelings out on others verbally”).

Because the capacity for abstract thought and complex meta-cognition undergoes considerable development during adolescence, alongside structural and functional changes in the brain (31), it would be important to ensure that self-report questionnaires designed to measure ER in young adolescents are as cognitively simple as possible. Meta-cognitive complexity can be conceptualized in terms of the number of “meta-cognitive relations” involved in an item (where cognitions are defined as thoughts with propositional content, and meta-cognitive relations are defined in terms of cognitions whose propositional content refers to other cognitions *i.e.*, “thoughts about thoughts”). For example, it may be argued that the CERQ item “I am preoccupied with what I think and feel about what I have experienced” contains two meta-cognitive relations, one nested within the other: 1) having thoughts about one’s thoughts (“I am preoccupied with what I think”); 2) thoughts which in turn are about the contents of one’s experiences (“about what I have experienced”). Such meta-cognitively complex items may be problematic, considering the individual differences in meta-cognitive capacity that can be expected in young adolescents.

### Current Study

In the present study we used a self-report questionnaire for measuring young adolescents’ ER strategies in daily life that was developed with the ambition of including only items that would not require complex meta-cognitive reasoning. This instrument was developed as part of a longitudinal project on “Deliberate self-harm, emotion regulation and interpersonal relations in youths” (SOL project) [for details see (39)], and the psychometric properties were established in a large sample of Swedish adolescents. The current study also aimed to elucidate the prospective associations between ER strategies, mental health problems, and self-injurious behaviors in adolescence and young adulthood.

## Materials and Methods

### Development of the Adolescents’ Emotion Regulation Strategies Questionnaire

The AERSQ asks participants what they do when they feel “sad, disappointed, nervous, afraid, or experience other negative or distressing feelings,” and presents them with a list of possible behaviors and ways of thinking, while asking them to estimate how often they engage in each of these on a 5-point scale ranging from 1 (never) to 5 (very often). We generated the items of the first version of the AERSQ partly on the basis of a review of existing questionnaires and partly on the basis of discussions among psychologists affiliated with the project as well as feedback from a group of adolescents who were given the questionnaire for comment. A 22-item version of the questionnaire was tested among 265 adolescents (137 girls and 128 boys) aged 14 and 15 years from six schools in southern Sweden (40). As a way to generate additional items for inclusion in the questionnaire, the adolescents who participated in the pilot study were also asked to give examples of what else they did in response to stressful emotions.

A factor analysis of these data led to the preliminary identification of four factors: rumination/negative thinking, distraction, positive reorientation, and communication (40). However, only the first two had a satisfactory internal consistency (*α* =.78 and *α* =.69, respectively). In four of the schools, test–retest data were available, with test–retest intervals varying from 44 to 126 days. In the school with the shortest inter-test interval (44 days), the test–retest correlations were large for three of the subscales—rumination/negative thinking (*r* =.80), distraction (*r* =.71), and communication (*r* =.74)—but only moderate for the positive reorientation subscale (*r* =.48). Thus, the test–retest reliability was good only for three subscales. Because the retests were not carried out until after 44 days, however, it is difficult to know whether the lower stability of the positive reorientation scale was due to measurement unreliability or actual alterations in participants’ behavior. Based on the results of this pilot study, we modified the questionnaire and added new items in line with adolescents’ responses, thus yielding the present 25-item version (see **Table 1** for an English translation of the items).

**Table 1:** Summary of the results of the exploratory factor analysis.

Item (in English translation)	Factor				
	1	2	3	4	5
***What do you do when you feel sad, disappointed, nervous, afraid, or experience other negative or unpleasant feelings****?*					
4. I think negative thoughts about myself	**.736**	−.105	.032	−.080	.115
14. I have the urge to physically hurt myself	**.675**	−.178	.088	−.076	.061
6. I think that others are more fortunate than me	**.671**	−.062	.022	.056	.029
5. I think that I am badly treated by others	**.579**	−.061	−.080	−.003	.063
13. I feel angry over having these feelings	**.571**	.092	.049	−.062	.075
3. I withdraw and keep to myself	**.530**	−.098	−.326	−.115	.077
9. I think that it is impossible to do anything about how I feel	**.547**	.010	.031	−.025	.024
10. I try to find the positive aspects of what has happened	−.171	**.629**	.128	.153	.071
8. I try to do something that will make me feel better	−.119	**.606**	.174	.140	−.002
11. I try to avoid thinking about my unpleasant feelings	−.027	**.524**	−.030	.022	.093
12. I try to think about pleasant things and daydream	−.063	**.449**	.112	.193	.232
25. I speak with friends on the phone	−.035	.084	**.727**	.197	.197
1. I speak with friends about how I feel	−.024	.198	**.703**	.030	.146
17. I eat something	.037	.073	.051	**.527**	.123
16. I listen to music or watch TV or online videos	.016	.128	.114	**.487**	.144
20. I write to or chat online with others	.018	−.018	.407	**.497**	−.014
22. I play video games or computer games	−.112	.000	−.261	**.487**	−.176
23. I sleep, rest, and relax	−.045	.216	.042	**.400**	.158
18. I read	.003	.167	−.023	.241	**.542**
19. I write a diary	.209	.015	.247	.032	**.482**
21. I draw, paint, play an instrument, or dance	.104	.147	.234	.140	**.443**
2. I speak with adults about how I feel	−.233	.279	.244	.056	.182
7. I think that it is best to accept how I feel	.345	.350	−.005	.050	−.039
15. I have the urge to physically or mentally hurt others	.315	−.080	−.075	.018	−.075
21. I go for a walk, cycling, work out, exercise, or partake in sports	−.118	.115	.179	.337	.120

Extraction Method: Principal Axis Factoring. Rotation Method: Varimax with Kaiser Normalization.

Rotation converged in six iterations.

### Participants

The SOL project involved three waves (2007, 2008 and 2017) of data collection. The original sample of 1,064 adolescents were all enrolled in Grade 7 and Grade 8 of regular schools in a municipality in southern Sweden with about 40,000 inhabitants in 2007. Of the students in this cohort, 991 (93%; mean [*SD*] age 13.7 [0.68]; 50.3% girls) participated in the data collection at Time 1 (T1). One year later (T2), when these students had entered Grades 8–9 (some students moving to the municipality after T1), another round of data collection was conducted among all eligible students (*N* = 1,098); in this round, 984 students participated (90%; mean [*SD*] age 14.8 [0.69]; 51.1% girls). The total number of eligible students at T1 and/or T2 was 1,109, and this became the target sample for the 10-year follow-up data collection (T3) in 2017. Of the individuals in this sample, 557 participated (response rate: 50.2%; mean [*SD*] age 25.3 [0.68]; 59.2% women).

### Procedure

At T1 and T2, the data were collected in collaboration with the municipal body of the selected area and each of the regular schools therein. Informed consent [using a passive consent procedure; for more details see (41)] was obtained from participating students and their parents before any data were collected. At both these time points, students completed the AERSQ and measures of self-injury and other psychological or interpersonal problems. All measures were administered in a classroom setting during ordinary lecture time by research assistants, who were either licensed psychologists or senior students in the psychologist program. The participants were asked to not write their names anywhere on the questionnaires to ensure confidentiality. Numeric codes were used to identify participants and to match the data from T1 and T2.

Ethical approval was obtained from the Regional Ethics Committee at Lund University in 2005 (for the data collections at T1 and T2) and 2016 (for the data collection at T3). In accordance with the ethical approval in 2005, we saved the list of participants’ codes for a future 10-year follow-up. To conduct the follow-up at T3, we sent participants’ names from the code lists from the prior two surveys to the Swedish state’s personal address register (SPAR) to identify their present locations. After receiving the current personal addresses of the participants, we sent letters describing the purpose and procedure of the follow-up to all potential participants. They were informed that their participation was voluntary, and were asked to complete a battery of questionnaires on ER abilities, self-harm, emotional distress and positive mental functioning, either *via* a paper-and-pencil form or a confidential web-survey designed using the Lund University survey system, Survey & Report. Numeric codes were used on all study documents throughout the study to identify participants in order to preserve their confidentiality. After completing the survey, participants received two cinema tickets or four lottery tickets as compensation.

### Measures

Besides the AERSQ, we administered the following measures at T1, T2, and/or T3.

#### Nonsuicidal Self-Injury at T1, T2 and T3

To measure nonsuicidal self-injury (NSSI), we used the 9-item shortened version of the Deliberate Self-Harm Inventory (DSHI-9r), which was modified from Gratz’s (42) original Deliberate Self-Harm Inventory (DSHI), and adapted to the Swedish population by Lundh, Karim, and Quilisch (43), Bjärehed and Lundh (40), and Lundh, Wångby-Lundh, and Bjärehed (44). The respondents were instructed to rate how often they had deliberately engaged in nine forms of self-injurious behavior (*i.e.*, cutting, minor cutting causing bleeding, burning, punching/banging oneself, biting, carving, severe scratching, sticking sharp objects into one’s skin, and preventing wounds from healing) over the past 6 months, on a scale from 0 (“never”) to 6 (“more than five times”). The DSHI-9 showed good test–retest reliability (40). The Cronbach’s alpha values for the DSHI-9r were .90 (T1), .89 (T2), and .81 (T3) in this study.

#### Psychological Problems and Strengths at T1 and T2

The Strengths and Difficulties Questionnaire–self-report version [SDQ-s; (45)] was used to measure adolescents’ psychological problems and strengths. The SDQ-s is a widely used screening instrument for psychological problems among children and adolescents, which contains five subscales with five items each: hyperactivity/inattention, emotional symptoms, conduct problems, peer problems, and prosocial behavior. Each item is rated on a 3-point scale (0 = not true, 1 = somewhat true, 2 = certainly true) within a timeframe of the previous six months. The Swedish version of the SDQ-s was empirically validated by Lundh, Wångby-Lundh, and Bjärehed (44). In the present study, the Cronbach’s alpha values of the five subscales were as follows: hyperactivity–inattention (T1: *α* =.66; T2: *α* =.66), emotional symptoms (T1: *α* =.67; T2: *α* =.69), conduct problems (T1: *α* =.57; T2: *α* =.60), peer problems (T1: *α* =.56; T2: *α* =.54), and prosocial behavior (T1: *α* =.68; T2: *α* =.70).

#### ER Abilities at T3

The Brief Difficulties in Emotion Regulation Scale [DERS-16; (46)] was used to evaluate young adults’ difficulties in ER, including lack of emotional clarity (*e.g.*, “I have difficulty making sense out of my feelings”), difficulties engaging in goal-directed behaviors (*e.g.*, “When I am upset, I have difficulty getting work done”), difficulties controlling impulses (*e.g.*, “When I am upset, I become out of control”), ineffective emotion regulation strategies (*e.g.*, “When I am upset, I believe that I will remain that way for a long time”), and non-acceptance of emotional responses (*e.g.*, “When I am upset, I feel ashamed with myself for feeling that way”). Participants estimate how often each of the 16 statements applies to them on a 5-point scale ranging from 1 (almost never) to 5 (almost always). The Cronbach’s alpha for the DERS-16 was .95 in this study.

#### Emotional Distress at T3

The Depression, Anxiety and Stress Scale [DASS-21; (47)] was used to evaluate participants’ emotional distress in young adulthood. The DASS-21 comprises three subscales: depression symptoms, anxiety symptoms, and stress/tension. Each subscale contains 7 items (*e.g.*, “I felt downhearted and blue” for depression; “I felt I was close to panic” for anxiety; “I found it hard to wind down” for stress/tension) rated on a 4-point scale ranging from 0 (never) to 3 (almost always). In this study, the Cronbach’s alpha values for the three subscales were as follows: .90 for depression, .79 for anxiety, and .87 for stress.

#### Positive Mental Functioning at T3

The Flourishing Scale [FS; (48)] and the Satisfaction with Life Scale [SWLS; (49)] were used to evaluate positive mental functioning. The FS is a brief 8-item measure of psychological and social well-being; it assesses the respondent’s self-perceived success in important areas such as relationships, self-esteem, purpose, and optimism. Participants indicate how much they agree or disagree with each of the 8 items (*e.g.* “I lead a purposeful and meaningful life”) using a 7-point scale (7 = strongly agree, 1 = strongly disagree). The total score ranges from 8 to 56. A higher score represents a person with many psychological resources and strengths. In this study, the Cronbach’s alpha for the scale was.88.

The SWLS is a measure of life satisfaction that contains five items (*e.g.* “I am satisfied with life”), each rated on a 7-point scale ranging from 7 (strongly agree) to 1 (strongly disagree). The Cronbach’s alpha for this scale was.92 in this study.

### Statistical Analysis

To examine the internal structure of the AERSQ, we used the T1 data to conduct an exploratory factor analysis (EFA; principal axis factoring with varimax rotation). Of the 993 participants included in the analysis, 883 participants had full data on the AERSQ at T1 and 100 had no more than three missing values on the AERSQ at T1. To compare participants with and without missing values, Little’s Missing Completely at Random (MCAR) test was conducted. Although the test was significant, *χ*^2^(868) = 961.47, *p* =.015, the normed *χ^2^ (i.e., χ^2^*/*df)* was 1.11; according to the guideline by Bollen (50), this value indicates that the pattern of missing data was not meaningfully different from a missing completely at random pattern. The missing values were imputed using the expectation–maximization (EM) algorithm before conducting the EFA.

Next, we used the T2 data to validate the adequacy of the best measurement model *via* confirmatory factor analysis (CFA). Likewise, only participants with full data (n = 898) and no more than 3 missing values (n = 81) on the AERSQ at T2 were included. Also as above, Little’s MCAR test was significant, *χ^2^* (928) = 1040.68, *p* =.006, but the normed *χ^2^* was 1.12 indicating that the pattern of the missing data was not meaningfully different from a missing completely at random pattern (50). Missing values were imputed before the CFA using the EM algorithm. The goodness-of-fit was assessed using the relative chi-square (chi-square to df ratio), comparative fit index (CFI), root mean square error of approximation (RMSEA), and standardized root mean square residual (SRMR). A chi-square to df ratio below 2 is preferred, but one between 2 and 5 is considered acceptable (51). A CFI should be equal to or greater than.90 to accept the model, indicating that 90% of the covariation in the data can be reproduced by the given model (52). As for RMSEA and SRME, 0 ≤ RMSEA ≤.05 and 0 ≤ SRMR ≤.05 indicate a good fit, while.05 < RMSEA ≤.08 and .05 < SRMR ≤ 0.10 indicate an acceptable fit (53).

The reliability of the AERSQ was tested by calculating the Cronbach’s *α* coefficient for each subscale at T1 and T2. Although .70 is recognized by many to be the arbitrary cut-off for an acceptable Cronbach’s *α* value, this cut-off has also been criticized in different articles. In a recent review, Taber (54) provided illustrative examples from the science education literature showing a wide range of values treated as acceptable or satisfactory (*e.g.* as low as *α* =.45) in different articles and also raised concerns with regards to the arbitrary value of .70 as a sufficient measure of an acceptable internal consistency of an instrument. In this study, we used .60 as the criteria for acceptable internal consistency in exploratory research recommended by Hair et al. (55).

Next, we evaluated the 1-year test–retest stability by calculating Pearson’s correlation coefficient between T1 and T2 scores for each subscale. Independent samples t-tests and t-tests for repeated measures were used to examine gender differences and the mean change in the AERSQ scores over one year for each gender, respectively.

The construct validity of the AERSQ was tested by calculating the correlations between the AERSQ subscales and the DERS-16.

To evaluate the external validity of the AERSQ, we calculated correlations between the AERSQ subscales and measures of NSSI, internalizing/externalizing problems, emotional distress, and positive mental functioning. Bonferroni corrections (56) were used to exclude spurious significant correlations due to type I errors.

The CFA using the Maximum Likelihood (MLR) estimation was conducted in Mplus 7.0 (57). All the other analyses were conducted using SPSS Statistics 25 (IBM Corp., Armonk, NY).

## Results

### Factor Analyses

Principal axis factoring with varimax rotation was conducted on the 25-item AERSQ at T1. The Kaiser measure of sampling adequacy was.823, making it well above the accepted cutoff of 0.6 and thus indicative of good factorability. Six factors had eigenvalues greater than 1; however, none of the items loaded above 0.40 on Factor 6 and, thus a five-factor solution was chosen. These five factors explained 48.6% of the variance, and all but four items had loadings higher than.40 on the intended factors. Further, one item (“I write to or chat online with others”) showed a cross-loading on Factors 3 and 4. While Factor 3 included items specifically concerning oral communication (speaking) with friends, the item “I write to or chat online with others,” despite relating to communication, had a higher loading on Factor 4, which included items measuring various forms of distraction; thus, it was included in Factor 4.

**Table 1** presents the item loadings on the five factors. The items loading on Factor 1 all concerned *rumination*/negative thinking, or repetitively thinking about one’s emotional distress and about the potential causes and results of that distress. The items loading on Factor 2 represented *positive reorientation*, or reinterpreting an emotional stimulus and finding positive meaning in it to alter its emotional influence. The items loading on Factor 3 represented *communication*, or explicitly expressing one’s emotional distress and communicating with others so as to reduce that distress. The items loading on Factor 4 represented *distraction*, referring to behaviors that divert one’s attention away from an emotional stimulus and towards other things. Finally, the items loading on Factor 5 represented *cultural activities*, or engaging in activities such as writing, reading, drawing, dancing, and making music in order to regulate emotions.

**Figure 1:**
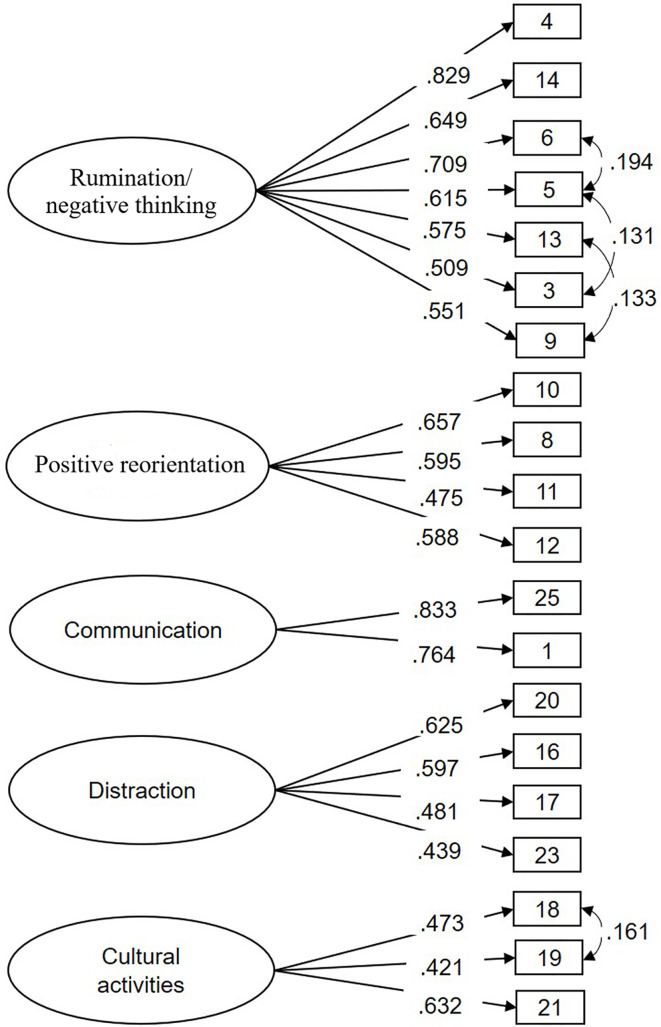
Standardized factor loadings in the CFA model.

Based on the EFA results, four items were dropped from the AERSQ. We then confirmed the five-factor model using a CFA with the remaining 21 items measured one year later, at T2. The CFI of 0.844 did not reach the cut-off value for acceptability, although the other indices were generally acceptable (χ^2^ = 854.15, df = 179, χ^2^/df = 4.77, RMSEA =.062, SRMR =.063). A closer look at the factor loadings revealed that one item (item 22 “I play video games or computer games”) had an exceedingly low loading (.122) on the intended factor (distraction); the factor loadings of all other items were higher than.45. Thus, item 22 was dropped from the CFA model. Moreover, based on the modification indices, we allowed some residuals among indicator variables belonging to the same factor, being conceptually related and also having the highest standardizes residual covariance to correlate (*i.e.*, item 3 with item 5, item 5 with item 6, item 9 with item 13, item 18 with item 19; see also **Figure 1**). These changes improved the fit of the model to an acceptable level, χ^2^ = 587.41, df = 156, χ^2^/df = 3.77, RMSEA =.053, SRMR =.053, and CFI =.895. **Figure 1** displays the final CFA model.

### Reliability, Stability, and Intercorrelations

**Table 2** shows the internal consistency coefficients (Cronbach’s *α*) and intercorrelations among the five subscales at T1 and T2 separately for girls and boys. The Cronbach’s *α* values were satisfactory overall (higher than or close to.60), with the rumination/negative thinking subscale having the highest values (T1: *α* =.81; T2: *α* =.83) and the cultural activities subscale having the lowest values (T1: *α* =.54; T2: *α* =.55). In addition, for the whole sample, the 1-year test–retest stability of the five subscales was as follows: *r* =.61 for rumination/negative thinking, *r* =.63 for communication, *r* =.37 for positive reorientation, *r* =.44 for distraction, and *r* =.61 for cultural activities.

**Table 2:** Internal consistency values (in the parentheses on the diagonal) and intercorrelations among the AERSQ subscales at T1 and T2 for girls (under the diagonal) and boys (above the diagonal).

	1	2	3	4	5	6	7	8	9	10
1. T1 Rumination/negative thinking	(.81)	−.08	−.08	−.04	.13**	.55***	−.13**	−.01	.00	.13**
2. T1 Communication	−.28***	(.71)	.24***	.40***	.28***	-−.03	.45***	.05	.24***	.10*
3. T1 Positive reorientation	−.30***	.33***	(.67)	.28***	.19***	.04	.12*	.31***	.15**	.15**
4. T1 Distraction	−.07	.32***	.30***	(.59)	.29***	.03	.24***	.14**	.37***	.13**
5. T1 Cultural activities	.01	.17***	.32***	.35***	(.54)	.14**	.15**	.08	.19***	.46***
6. T2 Rumination/negative thinking	.58***	−.23***	−.23***	−.05	−.00	(.83)	−.04	.06	.03	.24***
7. T2 Communication	−.17***	.64***	.18***	.25***	.05	−.25***	(.78)	.25***	.43***	.26***
8. T2 Positive reorientation	−.12*	.24***	.42***	.19***	.14**	−.29***	.34***	(.67)	.33***	.19***
9. T2 Distraction	−.03	.21***	.10*	.50***	.13**	−.08	.39***	.33***	(.62)	.34***
10. T2 Cultural activities	.10*	.08	.20***	.15***	.59***	−.01	.12*	.24***	.35***	(.55)

***p < .001, **p < .01, *p < .05

N = 895–983; 895 participants had full data on AERSQ at both T1 and T2.

As for the intercorrelations between the five subscales, positive correlations for both genders were found among positive reorientation, communication, distraction, and cultural activities at both T1 and T2. As reported in **Table 2**, the intercorrelations ranged from .17 (between communication and cultural activities) to .35 (between distraction and cultural activities) for girls and from .19 (between positive reorientation and cultural activities) to .40 (between communication and distraction) for boys at T1. Similar intercorrelations were found for both genders at T2. Interestingly, while rumination/negative thinking was significantly negatively related to communication and positive reorientation at both T1 and T2 for girls, these intercorrelations were not significant at either T1 or T2 for boys. For boys, however, significant positive correlations were found between rumination/negative thinking and cultural activities in addition to the positive correlations between cultural activities and communication, positive reorientation, and distraction that were found in both genders; this suggests that engaging in cultural activities has complex relations with other ER strategies for boys.

As shown in **Table 3**, the most endorsed AERSQ scales were communication (only among girls), positive reorientation, and distraction (both genders); however, the results showed that girls reported higher scores on all AERSQ subscales, except positive reorientation and distraction at T1, with the highest effect sizes being for communication and cultural activities at both time points. Moreover, while rumination/negative thinking scores increased significantly for girls over one year, *t*(456) = 4.33, *p* < .001, and cultural activities scores decreased significantly, *t*(456) = −3.32, *p* < .001, no significant changes were found for boys.

**Table 3:** Descriptive statistics and results of independent t-test for gender differences on the AERSQ at two time points.

AERSQ scales	M (SD)			*t*	*p*	Cohen’s d
	All	Girls	Boys			
T1 Rumination/negative thinking	2.14 (0.79)	2.34^#^ (0.85)	1.93 (0.65)	8.41	<.001	0.55
T1 Communication	3.13 (1.16)	3.63 (1.14)	2.62 (0.95)	15.15	<.001	0.94
T1 Positive reorientation	3.35 (0.85)	3.40 (0.86)	3.31 (0.83)	1.64	.101	0.11
T1 Distraction	3.37 (0.82)	3.41 (0.79)	3.34 (0.84)	1.44	.150	0.09
T1 Cultural activities	2.08 (0.91)	2.44^#^ (0.97)	1.73 (0.68)	13.30	<.001	0.86
T2 Rumination/negative thinking	2.24 (0.84)	2.50^#^ (0.84)	1.96 (0.76)	10.23	<.001	0.68
T2 Communication	3.15 (1.21)	3.63 (1.15)	2.64 (1.06)	13.39	<.001	0.90
T2 Positive reorientation	3.37 (0.86)	3.45 (0.85)	3.29 (0.86)	2.95	.003	0.20
T2 Distraction	3.37 (0.84)	3.46 (0.80)	3.28 (0.87)	3.18	.002	0.22
T2 Cultural activities	1.98 (0.90)	2.30^#^ (0.93)	1.65 (0.74)	11.63	<.001	0.78

^#^Endorsement of the rumination scale increased significantly, t(456) = 4.33, p < .001, while endorsement of the cultural activities scale significantly decreased, t(456) = −3.32, p < .001 for girls across one year, while no significant changes were found for boys.

### Construct and External Validity

Before the relationships between the AERSQ scales and the variables measured at T3 were studied, attrition analyses were conducted by comparing the responders (n = 541) and nonresponders (n = 529) at T3 on all studied variables at T1 and/or T2. Of the sociodemographic variables, significantly more women responded to the survey at T3 (T1 & T2: 51%, T3: 58.4%; *χ^2^*(1) = 29.30, *p* < .001). Of the variables studied in the present study, nonresponders scored significantly higher on the SDQ-s Hyperactivity/Inattention scale (T1: *t*(974) = 3.24, *p* < .01, Cohen’s *d* = 0.21), SDQ-s Conduct problems scale (T1: *t*(972) = 2.12, *p* =.03, Cohen’s *d* = 0.14), and significantly lower on the SDQ-s Prosocial behavior scale(T1: *t*(974) = -2.12, *p* =.03, Cohen’s *d* = 0.14). However, as the Cohen’s *d*s indicate, these differences were of low or very low magnitude.

As shown in **Tables 4** and **5**, significant positive relationships (after Bonferroni corrections) were found between the AERSQ rumination/negative thinking (both T1 and T2) scores and the DERS total score at T3 for girls, and between rumination/negative thinking score at T1 and the DERS total score at T3 for boys. Rumination/negative thinking showed the clearest relationships with the positive and negative aspects of psychological health at all three time points for girls, while for boys the results were mixed. As reported in **Tables 4** and **5**, rumination/negative thinking was found significantly positively correlated with NSSI and internalizing and externalizing problems at both T1 and T2 for both genders. Regarding the T3 variables, for girls, rumination/negative thinking (at both T1 and T2) was significantly related to both positive and negative aspects of mental health, while for boys, rumination/negative thinking measured at T1 was significantly and negatively related to life satisfaction and flourishing.

**Table 4:** Correlations between T1 AERSQ subscales and other studied variables assessed at T1, T2 and T3.

	T1 Rumination/negative thinking	T1 Communication	T1 Positive reorientation	T1 Distraction	T1 Cultural activities
T1 constructs	N = 954–977				
	Girls/Boys	Girls/Boys	Girls/Boys	Girls/Boys	Girls/Boys
NSSI	.50***/.35***	−.16***/.03	−.21***/−.16***	−.14**/.05	−.03/.09
Hyperactivity	.27***/.24***	−.05/.02	−.15***/−.12**	−.02/.09	−.11*/−.11*
Emotional symptoms	.55***/.45***	−.19***/−.02	−.13**/−.05	.01/-.01	.10*/.08
Peer problems	.41***/.28***	−.33***/−.21***	-.21***/−.10*	-.12*/-.04	.08/.11
Conduct problems	.39***/.29***	−.10*−.07	−.29***/−.25***	-.09/.04	−.06/−.09
Prosocial behavior	−.05/−.14**	.19***/.17***	.30***/.30***	.12**/11*	.21***/.09*
					
**T2 constructs**	N = 882–896				
NSSI	.35***/.20***	−.11*/.10*	−.18***/.03	−.15**/.07	.01/.04
Hyperactivity	.23***/.18***	−.02/.10*	−.12*/−.07	.01/.12*	−.05/−.11*
Emotional symptoms	.44***/.36***	−.21***/−.08	−.10*/−.01	.02/−.01	.11*/.04
Peer problems	.33***/.29***	−.29***/−.18***	−.11*/.02	−.07/−.04	.12**/.07
Conduct problems	.27***/.20***	.01/.02	−.20***/−.12*	.03/.05	−.08/−.09
Prosocial behavior	−.06/−.10*	.12*/.07	.21***/.15**	−.01/−.01	.11*/.10*
					
**T3 constructs**	N = 494–511				
NSSI	.19***/.13	−.10/−.02	−.09/v.07	−.09/−.04	−.01/.03
DERS-total	.29***/.27***	−.11/.09	−.18**/−.07	−.10/−.08	−.02/.06
Depression	.34***/.21**	−.20***/.07	−.17**/.01	−.12/−.08	−.03/.06
Anxiety	.33***/.16*	−.12*/.04	−.08/−.07	−.04/−.11	−.01/-.01
Stress	.33***/.20**	−.11/.06	−.13*/−.06	−.08/−.02	−.01/-.00
Life satisfaction	−.27***/−.29***	.21***/.06	.11/.14*	.09/.07	−.03/−.07
Flourishing	−.35***/−.27***	.28***/.08	.27***/.15*	.13*/.15*	.03/−.06

***p < .001, **p < .01, *p < .05.

N varies due to missing values for all variables except the AERSQ.

Significant correlation coefficients after Bonferroni correction are shown in bold. For comparisons with T2 constructs for girls and boys, the corrected p value is.05/30 =.0017; for comparisons with T3 constructs for girls and boys, the corrected p value is.05/35 =.0014.

**Table 5:** Correlations between T2 AERSQ subscales and other studied variables assessed at T2 and T3.

	T2 Rumination/negative thinking	T2 Communication	T2 Positive reorientation	T2 Distraction	T2 Cultural activities
T2 constructs	N = 959–972				
	Girls/Boys	Girls/Boys	Girls/Boys	Girls/Boys	Girls/Boys
NSSI	.45***/.40***	−.18***/.03	−.18***/−.05	−.23***/−.04	−.01/.24***
Hyperactivity	.27***/.22***	−.02/−.00	−.02/−.03	.07/.03	−.09/−.08
Emotional symptoms	.59***/.48***	−.16***/−.10*	−.05/.02	.05/−.04	.22***/.16***
Peer problems	.34***/.39***	−.35***/.−.21***	−.11*/−.05	−.10/−.11	.14**/.15**
Conduct problems	.29***/.29***	−.01/−.08	−.06**/**−.21***	.09/-.05	−.02/.01
Prosocial behavior	−.10*/−.08	.12**/.18***	.15**/.23***	−.01/.13**	.11*/.06
					
**T3 constructs**	N = 483–501				
NSSI	.14**/.08	−.03/−.03	.01/−.08	−.01/−.09	.10/−.07
DERS-total	.36***/.15*	−.12/−.21**	−.13*/−.05	−.06/.01	.03/−.05
Depression	.34***/.13	−.17**/−.16*	−.13*/.01	−.01/−.02	−.01/−.05
Anxiety	.33***/.11	−.08/−.14*	−.08/.00	−.04/.09	−.04/−.07
Stress	.34***/.08	−.14/−.15*	−.09/−.10	−.06/.01	−.02/−.10
Life satisfaction	−.27***/−.12	.25***/.23***	.13*/.10	.06/.08	−.05/.07
Flourishing	−.28***/−.17*	.27***/.26***	.23***/.12	.03/.10	−.01/.03

***p < .001, **p < .01, *p < .05.

N varies due to missing values on variables except the AERSQ.

Significant correlation coefficients after Bonferroni correction are shown in bold. For comparisons with T2 constructs for girls and boys, the corrected p value is.05/30 =.0017; for comparisons with T3 constructs for girls and boys, the corrected p value is.05/35 =.0014.

As shown in **Tables 4** and **5**, the AERSQ communication subscale showed significant negative relationships after Bonferroni corrections with NSSI (for girls at T1 and T2), emotional problems (for girls at T1 and T2), peer problems (for both genders at T1 and T2), and depression (for girls at T1), as well as significant positive relationships with prosocial behavior (for both genders at T1, only for boys at T2), life satisfaction and flourishing (for girls at T1 and for both genders at T2).

As for the AERSQ positive reorientation subscale, significant negative relations after Bonferroni corrections were found with NSSI (for both genders at T1, only for girls at T2), hyperactivity (for girls at T1), peer problems (for girls at T1), and conduct problems (for both genders at T1, only for boys at T2). Significant positive relations were found between positive reorientation and prosocial behavior (for both genders at T1, only for boys at T2) and flourishing (T3 for girls).

For AERSQ distraction subscale, the correlations were weaker and the only significant negative correlation after Bonferroni correction was found between distraction and NSSI at T2 for girls. However, no clear longitudinal relationships were found between distraction (T1 or T2) and the variables assessed at T3.

The correlations between the AERSQ cultural activities subscale and the other variables were more unexpected, especially for boys. While this subscale was significantly and positively correlated with prosocial behavior for girls at T1, the scale was significantly and positively correlated with emotional problems (for both genders) and NSSI (for boys) at T2.

## Discussion

In the present study, we presented the psychometric properties of a self-report measure AERSQ developed to assess adolescents’ commonly used ER strategies in daily life. We used three-wave longitudinal data from a large cohort of Swedish adolescents to evaluate the internal structure, reliability, and validity of this AERSQ.

The factor analyses supported a five-factor structure for the AERSQ: rumination/negative thinking, positive reorientation, communication, distraction, and cultural activities. In terms of the internal consistency of the finalized AERSQ subscales, three out of the five subscales—Rumination/negative thinking, Communication, and Positive reorientation—showed very good or good acceptable internal consistency. The two remaining scales (Distraction and Cultural activities) had lower internal consistency and may need further revision.

The one-year test–retest stability coefficients for the five subscales were all above 0.60, except for positive reorientation (*r* =.37) and distraction (*r* =.44). These coefficients are similar to those reported by Gullone and Taffe (27), who also examined the 12-month stability of emotion regulation measured with the adapted ERQ-CA in adolescent samples. Although two retest coefficients could be perceived as rather low, this result is not surprising when considering that emotion regulation develops substantially throughout adolescence and becomes more trait-like with increasing age (58).

The five factors of the AERSQ correspond to the different distinctions between ER strategies made in the existing literature. While rumination/negative thinking and positive reorientation are cognitive strategies, the other three strategies regulate emotions through behavior. All these strategies, except communication, exploit intrapersonal resources to regulate emotions, while communication regulates emotions through interpersonal resources.

With respect to the external validity, we examined the associations between the AERSQ subscales and different mental health measures across three time points. Overall, the findings indicate that the cognitive strategies (especially rumination/negative thinking) have clearer and stronger relationships with different aspects of mental health than do the behavioral strategies distraction and cultural activities, especially prospectively. These findings are in line with those reported by Rood et al. (59). In their meta-analysis comparing the effects of rumination and distraction on depressive symptoms in a nonclinical sample of youth, Rood et al. found that there were significant and stable effects of rumination on concurrent and future levels of depression, but no significant effects for distraction. Furthermore, many studies have demonstrated, both cross-sectionally and longitudinally, close relationships between rumination and the increased risk and severity of a number of mental disorders and other aspects of psychological malfunctioning, such as impaired interpersonal relationships, poor academic and occupational performance [see a review in (6)]. However, limited research to date has directly addressed the reason individuals utilize such a detrimental cognitive style.

Positive reorientation, another cognitive ER strategy assessed by the AERSQ, showed significant negative correlations with some of the studied negative constructs, such as NSSI and conduct problems, for both genders at T1, as well as significant positive correlations with prosocial behavior at T1 and T2 and positive mental functioning at T3. Although these results were in line with the literature, the correlations were somewhat lower and the results more mixed compared to those found for the Rumination/negative thinking scale. Still, this ER strategy was one of the most endorsed strategies among girls and boys in our sample at both T1 and T2. According to Gross’s process model of ER (3, 16), positive reorientation (*i.e.*, positive reappraisal) intervenes in the emotion-generation process—specifically, individuals can negotiate stressful situations by taking an optimistic attitude, reinterpreting those stressful situations and finding positive meanings, and making active efforts to repair negative moods. Positive reorientation modifies not only what individuals think and feel inside, but also what they express and how they explicitly behave. Individuals who habitually use positive reorientation to regulate emotions have been found to be more likely to experience positive emotions; share emotions with friends; have fewer mental distress symptoms; and have greater self-esteem, life satisfaction, and other positive outcomes (21).

The AERSQ communication subscale represents the strategy of regulating emotions through drawing on interpersonal resources. Interestingly, this ER strategy was predominantly endorsed by girls. Our results showed robust relations between the communication subscale and other mental health indicators involving interpersonal functioning, being negatively associated with peer problems and positively associated with prosocial behavior. AERSQ communication scores at both T1 and T2 were also found to be significantly and positively related with positive mental functioning (*i.e.*, life satisfaction and flourishing) for girls at T3. It should be noted, however, that the communication subscale comprised only two items, which may not provide a complete measure of behaviors relevant to this strategy. To improve this particular subscale, extra items might need to be generated and incorporated in the future.

As for the distraction subscale, although it showed positive correlations with positive mental health indicators (*e.g.*, flourishing) and negative correlations with negative indicators (*e.g.*, NSSI), after Bonferroni correction none of these correlations were significant except for a negative correlation with NSSI for girls at T2. Furthermore, no clear longitudinal relationships were found between distraction (T1 or T2) and mental health indicators at T3. Previous research on the functions of distraction as an ER strategy has yielded mixed findings. On the one hand, distraction (which involves intentional deployment of attention away from negative emotional stimuli towards other things) is seen as a form of active problem solving (36), and has been shown to be an adaptive ER strategy in various studies [*e.g.*, (37, 60)]. On the other hand, several studies have not found any putatively beneficial effects of distraction on emotional distress symptoms [*e.g.*, (61)]. One study (62) found, in both a nonclinical sample and a clinical sample, that distraction can be either adaptive or maladaptive depending on whether it is combined with acceptance or avoidance strategies. In other words, it is adaptive when combined with active acceptance but maladaptive when combined with avoidance. Moreover, some scholars (63) have posited that distraction might have advantages in the short run but adverse outcomes in the long run. These conflicting results suggest that further research is needed to clarify the conditions under which distraction is functional or dysfunctional.

Finally, the relationships between cultural activities and other mental health indicators were more unexpected, especially among boys. This ER strategy was the least endorsed strategy by both boys (at T1 and T2) and girls (at T2). Previous research has shown that engaging in culture activities (*e.g.*, making music, writing, dancing, and crafts) might affect emotions through several different paths: it might function as a means of avoidance; it might help facilitate emotional discharge *via* “mental work”; or it might facilitate self-development, including self-identity, self-esteem, and agency (64). It is therefore possible that cultural activities in a broader sense (*e.g.*, including reading and writing a diary, as in the present study) could also have complex functions.

As described above, the correlational patterns between ER strategies and mental health indicators as well as the endorsement of different AERSQ scales differed between boys and girls, with the largest mean differences being for communication, cultural activities, and rumination/negative thinking. These results are in line with those reported in a meta-analytic review by Tamres, Janicki, and Helgeson (65), wherein consistent gender differences were found across studies in the strategies involved in verbal expressions to others or the self—to seek emotional support, ruminate about problems, or engage in positive self-talk, with females reporting more frequent usage of these strategies. Zimmermann and Iwanski (66) also found that females score significantly higher on social support seeking and dysfunctional rumination. Tamres, Janicki, and Helgeson (65) suggested that biological sex differences in responses to stress, along with gender socialization, are possible explanations for these findings. For example, women generally possess higher levels of the pituitary hormone, oxytocin, than do men. During times of stress, the release of oxytocin is related to downregulation of the sympathetic nervous system and facilitation of the parasympathetic nervous system, which is related to a “tend-and-befriend” response rather than a “fight-or-flight” response. Therefore, females are more likely than are males to seek out the support of others in time of stress, while males are more likely to use the avoidant or withdrawal strategies (67). As for gender socialization (68), women might be more socialized to seek out others for emotional support and express their feelings to others, whereas men tend to be discouraged from expressing their feelings to others, especially their feelings about life problems (69). Tamres, Janicki, and Helgeson (65) further suggested that expressions of feelings to others are likely to foster connections among women but might be viewed by men as revealing weaknesses and exposing vulnerabilities.

### Limitations and Future Directions

Methodologically, an important asset of the present study is its longitudinal design, which allowed for examination of the prospective relationships between the AERSQ strategies assessed in adolescence and ER abilities and other mental health indicators assessed in young adulthood. However, this study has some limitations that should be taken into consideration.

First, the present study was correlational, and correlations are always open to alternative causal explanations. Strictly speaking, we cannot conclude from correlational data that some emotion regulation strategies are adaptive and others maladaptive. As already noted, a given ER strategy might be adaptive or maladaptive depending on the context. Further, even if consistent positive correlations between the use of a specific strategy (*e.g.*, rumination/negative thinking) and future distress are found, this might have several possible explanations. It may, for example, be due to this strategy causing distress; to the fact that adolescents who are already in distress might make more use of this strategy; or to the fact that both are the result or a part of more basic phenomena. Likewise, negative associations between the use of one specific strategy (*e.g.*, positive reorientation) and future distress might be due to the fact that this strategy leads to less distress; to the fact that adolescents who feel less distress from the beginning make more use of this strategy; or to the fact that both are the result or a part of more basic phenomena.

Second, since two subscales (*i.e.*, Distraction and Cultural activities) showed low internal consistency and the CFA indicated some potential fit problems, the scale needs further revision and development. Although scales with low internal consistency might prove valid and useful (70), we cannot rule out that the low internal consistency of these two subscales might have led to inconsistent correlations over time.

Third, the present cohort was from the general population, and the established relationships in this study might be different in adolescents with diagnosed psychopathology. Therefore, future research should examine the validity of the AERSQ in clinical samples.

Fourth, this instrument was not developed through a “top-down” approach—that is, through theoretically derived categorization of five different ER strategies—but through a more inductive process based on what an adolescent is likely to do when faced with disturbing emotions. Adolescents who participated in the pilot study were also asked to add examples of what they would do in response to stressful emotions. One limitation of this procedure is that some forms of ER that could have been deduced from prior theory and research might have been missed; for example, expressive suppression is a widely studied strategy also used by adolescents, but this is not represented in the AERSQ.

Fifth, it was not easy to find wholly appropriate labels for all factors. For example, although Factor 1 was labeled Rumination/negative thinking, it also included an item that refers more to behavior than to thinking: “I withdraw and keep to myself.” Although affirmation of this item might be interpreted as an expression of negative thinking, it is not a direct example of it. Also, it would have been helpful to study the correlations between this factor and a validated measure of rumination. One might also question whether Cultural activities is a wholly appropriate name for Factor 5, given that one of the items is “writing a diary.” However, if “cultural” is defined according to some varieties of cultural anthropological thinking as activities that involve the use of human-made symbols, artifacts, and other human expressions, it may well be argued that writing a diary qualifies as a cultural activity along with reading, drawing, painting, dancing, and playing instruments.

Sixth, it is unclear as to whether our goal of avoiding meta-cognitive complexity in the AERSQ was entirely successful. For example, some degree of meta-cognitive complexity might be involved in two items: “I think that it is impossible to do anything about how I feel”, and “I think that it is best to accept how I feel”. Further, although the degree of meta-cognitive complexity of scale items might be measured *via* a pure textual analysis, it might also be of interest to test such items by asking adolescents how they interpret them.

To summarize, although the AERSQ showed good test–retest reliability and predictive validity over a 10-year period, it clearly has some limitations and therefore the version of the AERSQ studied herein might benefit from further development in terms of the included items and psychometric testing. We also think that a self-report instrument of this kind, which was designed to minimize meta-cognitive complexity, has the potential to contribute to greater knowledge of adolescents’ ER strategies and the association of such strategies with psychological functioning.

## Data Availability Statement

The datasets generated for this study are available on request to the corresponding author.

## Ethics Statement

The studies involving human participants were reviewed and approved by Regional Ethics Committee at Lund University. Written informed consent to participate in this study was provided by the participants’ legal guardian/next of kin.

## Author Contributions

DD, L-GL, and MW-L designed the larger project within which this study was conducted and wrote the protocol for it. YZ, DD, and AR conducted the statistical analyses. YZ wrote the first full draft of the manuscript. All authors worked on several edits of the paper. All authors contributed to and have approved the final manuscript.

## Funding

This study was funded by a grant from the Swedish Research Council for Health, Working Life and Welfare (2016-00248); by the Wenner-Gren Foundations; by the Thora Ohlsson Foundation; and by the Lundh Research Foundation UPD2017-0092, UPD2018-0187).

## Conflict of Interest

The authors declare that the research was conducted in the absence of any commercial or financial relationships that could be construed as a potential conflict of interest.
